# Association between Anxious and Depressive Symptomatology and Sexual Activity in Spain: A Cross-Sectional Study during the COVID-19 Quarantine

**DOI:** 10.3390/ijerph19010147

**Published:** 2021-12-23

**Authors:** Alejandro Gil-Salmerón, Guillermo F. López-Sánchez, Rubén López-Bueno, Shahina Pardhan, Igor Grabovac, Lee Smith

**Affiliations:** 1 International Foundation for Integrated Care, Oxford OX2 6UD, UK; 2 Vision and Eye Research Institute, School of Medicine, Faculty of Health, Education, Medicine and Social Care, Cambridge Campus, Anglia Ruskin University, Cambridge CB1 1PT, UK; shahina.pardhan@aru.ac.uk; 3 Department of Physical Medicine and Nursing, University of Zaragoza, 50009 Zaragoza, Spain; 4 Centre for Health, Performance and Wellbeing, Anglia Ruskin University, Cambridge CB1 1PT, UK; Lee.Smith@aru.ac.uk; 5 Centre for Public Health, Department of Social and Preventive Medicine, Medical University of Vienna, 1090 Vienna, Austria; igor.grabovac@meduniwien.ac.at

**Keywords:** COVID-19, lockdown, sexual health, mental health, anxiety, depression

## Abstract

Introduction: Evidence on sexual behaviour and COVID-19 shows a change in sexual habits; however, there is no research on the association between mental health and sexual activity. Aim: To examine the relationship between mental health and sexual activity during the quarantine in Spanish adults. Methods: A sample of 305 adults filled out an online questionnaire. Sexual activity was assessed with one question. Anxiety and depression symptoms were assessed using the Beck Anxiety Inventory (BAI) and the Beck Depression Inventory (BDI), respectively. To check associations between levels of both anxiety and depressive symptoms (exposure) and weekly prevalence of sexual activity (outcome), we conducted multiple logistic regression adjusted for control variables (marital status, employment, average household annual income, place of living, pre-COVID-19 sexual activity, current smoking, current alcohol consumption, chronic physical conditions, chronic psychiatric conditions, physical symptoms, and days of confinement). Results: Higher depression level was associated with lower weekly sexual activity in a dose-response fashion in the three implemented models. Participants with higher levels of depression were associated with significantly lower sexual activity in the fully adjusted model (OR: 0.09, 95% CI 0.01–0.61). Mild anxiety-level participants consistently presented significantly lower ORs for lower sexual activity than their minimal-anxiety category counterparts. Particularly, the fully adjusted model showed the lower values (OR: 0.40, 95% CI 0.19–0.84). Conclusion: The results of this study support existing evidence stressing the association between mental health and sexual activity in quarantined adults.

## 1. Introduction

As in many other countries, the Spanish Government has been implementing different public health strategies in order to slow the spread and “flatten the curve” of the ongoing human-to-human transmission of the COVID-19. In this regard, after the statement of the outbreak of COVID-19 as a global pandemic by the World Health Organisation and due to the high number of diagnosed cases in Spain, from 15 March until 2 May 2020, a period of total quarantine was established in Spain [1]. 

The health crisis and the restrictions imposed prompted a drastic situation for everyone with radical changes in daily life [2,3]. Among the different collateral impacts due to the pandemic situation and the public health measures to combat COVID-19 infection, sexual behaviour is one of the main concerns and it is being studied internationally [4,5,6]. This is because sexuality is an important element of human life and, according to The World Health Organisation, sexual health is “a state of physical, emotional, mental and social well-being related to sexuality; not merely the absence of disease, dysfunction or infirmity”. Consequently, a frequent and trouble-free sex life is associated with a plethora of physical and mental health benefits [4].

First studies on sexual behaviour and COVID-19 pointed out that there has been a change in sexual habits; in this regard, the lockdown has affected the sexual activity between cohabitants due to the difficulty of finding a moment of intimacy. As for the stable relationships who were not living together, the lockdown has made the physical contact impossible. Finally, for those looking for occasional sexual relations, the lockdown has created a drastic barrier for meeting a sexual partner [5]. Studies have suggested some changes based on the relationship status and the availability for living together during the implementation of movement control measures during the COVID-19 pandemic [6]. In this regard, there is evidence that all these changes have supposed that the quality of sex and satisfaction has decreased significantly [5,6]. Furthermore, Spanish women have been identified as a target group for promoting healthy regular practices regarding sex during COVID-19 quarantine since they were observed to reduce sexual activity during that period [7].

Scientific evidence shows that sexual inactivity is significantly related to different mental health conditions [8]. Moreover, it has been found that sexual dysfunction might lead to interpersonal conflicts by deteriorating either self-esteem or partner relationships [9]. On the other hand, there is evidence that positive mental health outcomes for males and females are significantly related to successful sexual activity. In this regard, psychological well-being by improving mood, even in depressed and high-anxiety patients [10,11], is positively related to trouble-free sexual activity.

To our knowledge, there is no evidence explaining how mental health affected the sexual activity status of the Spanish population during the quarantine. Therefore, this study aims to explain the association between depression/anxiety and sexual activity during quarantine among this population. 

## 2. Materials and Methods

### 2.1. Study Design and Participants

A cross-sectional online survey was administered during the COVID-19 quarantine in Spain. This study followed the principles of the World Medical Association Declaration of Helsinki and was approved by the Ethics Committee of Research in Humans of the University of Valencia (1 April 2020; register code 1278789). The data collection comprised one month (from 1 April 2020 to 1 May 2020). On 9 May 2020, the gradual opening in Spain commenced [12]. 

Spanish adults aged 18 years and over (i.e., the age range of those who completed the survey 18–74 years) that confirmed to be self-isolated due to the COVID-19 pandemic were eligible to participate. A convenience sample of participants were recruited through social media (e.g., Facebook, Twitter, Whatsapp). They were directed to a data encrypted website where they indicated their consent to participate after reading an information sheet, and confirmed that they were in a quarantine situation. Overall, a participation rate of 42% was reached. The data provided were anonymous and treated accordingly to Spanish law regarding general data protection. This manuscript was written in accordance with the STROBE Statement (Strengthening the Reporting of Observational Studies in Epidemiology) [13]. 

### 2.2. Key Measures

#### 2.2.1. Sexual Activity (Exposure)

In the participant survey, sexual activity was defined as sexual intercourse, masturbation, petting, or fondling. Participants were asked, “On average, after self-isolating/social-distancing, how many times have you engaged in sexual activity weekly?”

#### 2.2.2. Anxiety (Outcome)

Anxiety symptoms were assessed using the Spanish version of the Beck Anxiety Inventory (BAI) [14], which has shown high consistency (Cronbach alpha coefficient = 0.93) and moderate correlation (r = 0.32) with the Trait-Anger scale of the STAXI 2 [15] The BAI is composed of 21 items. Each item consists of a different anxiety symptom in which the participant scores how he/she felt in relation to that symptom during the last month, on a scale that varies from 0 (Not at all) to 3 (Severely, it bothered me a lot). The overall scores range from 0 to 63. Scores ranging from 0–7 indicate minimal/no anxiety symptoms; 8–15 indicate mild anxiety symptoms; 16–25 indicate moderate anxiety symptoms, and 26–63 indicate severe anxiety symptoms [14]. For this study, the group of severe anxiety was underrepresented with 4 participants, so they were excluded from the analysis. 

#### 2.2.3. Depression (Outcome)

Depression symptoms were assessed using the Spanish version of the Beck Depression Inventory (BDI) [16]. This has shown high consistency (Cronbach alpha coefficient = 0.83) [17]. The BDI is composed of 21 items. Each item consists of a series of four statements based on the severity of depression symptoms. The score of each item varies from 0 (minimum score) to 3 maximum score). The overall score of the instrument range from 0 to 63. Scores ranging from 0–13 indicate minimal/no depression; 14–19 indicate mild depressive symptoms; 20–28 indicate moderate depressive symptoms, and 29–63 indicate severe depressive symptoms [16,18].

#### 2.2.4. Covariates

Collected demographic data were conducted through single-item questions: “What is your gender?” and potential answers comprising “male” or “female”, “What is your Age?”, and potential answers including 10-year age bands, “What is your marital status?”, with potential answers including “single”, “divorced”, “separated”, “widowed” or “married/in a domestic partnership”, “What is your current status?”, and possibilities comprising “employed”, and “not employed”, “What is your average household annual income?”, and possible answers including “<€15,000”, “€15,000–<€25,000”, “€25,000–<€40,000”, “€40,000–<€60,000”, “≤€60,000”. Participants were also asked whether they were residing in the Iberian Peninsula or other Spanish regions outside the Iberian Peninsula (“What part of the country do you live in?”), and possible answers comprising “Peninsula”, “Balearic Islands”, “Canary Islands”, and “Ceuta or Melilla”. Measures of health status included whether respondents were a current consumer of alcohol (“Do you drink alcohol?”), and smoker (“Do you smoke?”), and possible answers for these two questions comprising “yes”, or “no”; “Have you ever been diagnosed by a health professional with: (tick all that apply)”: “hypertension”, “obesity”, “myocardial infarction”, “angina pectoris and other coronary diseases”, “other cardiac diseases”, “varicose veins of lower extremities”, “osteoarthritis”, “chronic neck pain”, “chronic low back pain”, “chronic allergy (excluding allergic asthma)”, “asthma (including allergic asthma)”, “chronic bronchitis”, “emphysema or chronic obstructive pulmonary disease (COPD)”, “type 1 diabetes”, “type 2 diabetes”, “diabetic retinopathy”, “peptic ulcer disease”, “cataract”, “urinary incontinence or urine control problems”, “hypercholesterolemia”, “chronic skin disease”, “chronic constipation”, “liver cirrhosis and other hepatic disorders”, “stroke”, “chronic migraine and other frequent chronic headaches”, “hemorrhoids”, “cancer”, “osteoporosis”, “thyroid disease”, “injury”, and “renal disease”, as well as the option to declare “other psychiatric conditions”. Moreover, participants were asked if they had experienced any physical symptom of COVID-19 during the confinement: “What guidelines are you following for self-isolation?”, and responses comprising the selection of one or more of the following symptoms: “high temperature”, “persistent cough”, “sore throat”, and “runny nose”. Finally, the participants were asked for the number of days they had been confined (“What day of self-isolation are you currently on?”).

### 2.3. Statistical Analyses

The statistical analyses were performed with Stata v16.1. (StataCorp, TX, USA) Participants with and without regular sexual activity were compared regarding socioeconomic characteristics, health characteristics, and other specific characteristics concerning COVID-19 confinement using chi-squared tests for categorical variables and *t*-tests for continuous variables. The cut-off point for age was set at 45 years old since it is a turning point concerning mental health for Spanish men and women [19]. Estimations for effect sizes were conducted using phi coefficient (chi-squared tests with binary categorical variables), Cramer’s V (chi-squared tests with categorical variables with more than 2 categories), and Cohen’s d (*t*-tests with continuous variables). To check associations between weekly prevalence of sexual activity among different levels of both depression and anxiety, we conducted multiple logistic regression adjusted for control variables (marital status, employment, average household annual income, place of living, pre-COVID-19 sexual activity, current smoking, current alcohol consumption, physical conditions, psychiatric conditions, physical symptoms, and days of confinement). There were less than 3.5% of missing values for the variables used in this study, thus, complete case analysis was carried out. The level of statistical significance was set at *p* < 0.05.

## 3. Results

A total of 305 Spanish confined adults completed the survey. Overall, 85 (27.9%) males and 220 females (72.1%) participated. The age distribution was: 18–44 years (*n* = 277), 45–74 years (*n* = 28). Significant differences between participants regarding marital status, employment, depression, anxiety, and sexual activity before the COVID-19 confinement were identified. The rest of the sample characteristics are shown in Table 1. 

The association between the level of depression and sexual activity is displayed in Table 2. Higher depression level was associated with lower sexual activity in a dose-response fashion in the three implemented models. Participants with higher levels of depression were associated with significantly lower sexual activity in the fully adjusted model (OR: 0.09, 95% CI 0.01–0.61). Also, Table 3 shows the association between anxiety and sexual activity, in which mild anxiety level was associated with lower sexual activity (OR: 0.40, 95% CI 0.19–0.84; fully adjusted model). 

## 4. Discussion

The main objective of this study was to investigate the association between mental health and sexual activity during quarantine for adults in Spain. In the present sample, comprising 305 Spanish confined adults, we found that both higher anxiety and depressive symptoms had a negative dose-response on the level of sexual activity. 

The finding that sexual activity is associated with worse mental health outcomes is important and supports and adds to previous evidence [10,11]. Indeed, the present study found that people who have higher levels of depression have less sexual activity during the quarantine. Additionally, we found that the participants who have higher levels of anxiety presented a significantly lower chance to have sexual activity. Taken together, the present findings suggest that better mental health status, having less anxious and depressive symptomatology, is associated with having a more active sexual activity during COVID-19 quarantine. 

Moreover, the COVID-19 pandemic is having an impact on the sexual life of the population [20,21]. There is evidence showing a reduction and different changes in sexual activity due to the fear of how contagious COVID-19 is, as well as the consequences of the lockdown, such as loss of privacy and the incapacity of meeting sexual partners [22,23]. This reduction of sexual activity might lead to a decrement of the level of physical activity, which, in turn, could worsen mental health [24]. In this regard, physical activity was significantly associated with lower perceived anxiety and lower perceived worse mood in a large sample of Spanish confined adults [25]. Similar evidence has been found between depression and anxiety symptoms and general physical activity in a sample of adults aged 18 years and over, residing in UK and social-distancing (i.e., following UK government enforced restrictions that limited movement of people) due to COVID-19 [26].

Other studies have shown that the COVID-19 pandemic had a wide range of psychological impact on the Spanish population, and this might affect the predisposition and the mood for having personal intercourse [27]. Different studies have shown that the uncertainty of the COVID-19 situation, as well as its development and consequences, have led to worse mental health outcomes such as anxious and depressive symptomology [27,28]. For that reason, the promotion of protective factors for mental health could lead to the development of tailored strategies focusing on physical activity and an active and healthy sexual life during the whole cycle of life, due to the potential relationship of these variables. Another implication, also highlighted in another study, should be the need to focus on enhancing sexual wellness by educating on and supporting sexual activities during the implementation of movement control measures [29].

The main strength of this study is that it is the first study reporting the association between mental health and sexual activity in a sample of quarantined adults. Another strength of the present study is the use of two well-known valid and reliable inventories to assess mental health: Beck Anxiety Inventory (BAI) and Beck Depression Inventory (BDI). However, the present findings must be interpreted in light of the study limitations. First, sexual activity was self-reported, potentially introducing self-reporting bias into the findings. Second, due to the method of sampling (convenience sampling), there is the possibility of a selection bias. Third, analyses were cross-sectional, and, thus, it was not possible to determine the direction of the association. Moreover, given the critical and unprecedented situation, we avoided asking too many questions, thus, important information, such as the number of days confined was not collected. Finally, more than 70% of the sample are women, thus, a selection bias regarding gender is plausible. Similarly, because the option of binary gender was not given to the participants, there is still a chance for some misclassification bias. 

## 5. Conclusions

In conclusion, in the present sample of confined Spanish adults, those who had more anxious and depressive symptomatology presented less sexual activity during the quarantine. To our knowledge, this is the first study on mental health and sexual activity in a sample of quarantined adults. In this regard, mental health outcomes and sexual activity seem to be associated, for that reason, future research must focus on inferring the direction of this association. 

## Figures and Tables

**Table 1:** Sample characteristics (overall and by sexual activity status).

Characteristics	Category	Overall (*N* = 305)	Sexual Activity during COVID-19 Confinement		Effect Size ^a^	*p* Value ^b^
			No (*N* = 94)	Yes (*N* = 211)		
Sex	Male	27.9	17.7	82.3	0.17	0.002
	Female	72.1	35.9	64.1		
Age	18–44 years	90.8	30.3	69.7	0.03	0.556
	45–74 years	9.2	35.7	64.3		
Marital status	Single/separated/divorced/widowed	71.5	37.2	62.8	0.22	0.000
	Married/in a domestic partnership	28.5	14.9	85.1		
Employment	No	34.4	42.9	57.1	0.19	0.001
	Yes	65.6	24.5	75.5		
Annual household income	Less than 15,000€	55.4	34.3	65.7	0.17	0.066
	15,000€ to 25,000€	31.2	30.5	69.5		
	25,000€ to 40,000€	10.8	15.2	84.8		
	40,000€ to 60,000€	1.6	0.0	100.0		
	More than 60,000€	1.0	66.7	33.3		
Living outside the peninsula (Balearic Islands, Canary Islands, Ceuta or Melilla)	No	84.6	32.2	67.8	0.07	0.231
	Yes	15.4	23.4	76.6		
Current smoking	No	15.7	29.2	70.8	0.02	0.787
	Yes	84.3	31.1	68.9		
Current alcohol consumption	No	41.0	24.8	75.2	0.11	0.058
	Yes	59.0	35.0	65.0		
Any symptoms related to COVID-19	No	96.4	29.9	70.1	0.10	0.083
	Yes	3.6	54.5	45.5		
Depression	Minimal (0–13 points)	73.1	24.7	75.3	0.23	0.001
	Mild (14–19 points)	14.1	44.2	55.8		
	Moderate (20–28 points)	10.2	48.4	51.6		
	Severe (29–63 points)	2.6	62.5	37.5		
Anxiety	Minimal (0–7 points)	68.5	24.4	75.6	0.25	0.000
	Mild (8–15 points)	22.0	44.8	55.2		
	Moderate (16–25 points)	8.2	36.0	64.0		
	Severe (26–63 points)	1.3	100.0	0.0		
Any chronical physical condition	No	46.9	28.7	71.3	0.04	0.445
	Yes	53.1	32.7	67.3		
Sexual activity before the COVID-19 confinement	No	11.8	97.2	2.8	0.53	0.000
	Yes	88.2	21.9	78.1		
Number of days of confinement	Mean (Standard Deviation)	30.1 (8.0)	29.4 (7.2)	30.5 (8.3)	0.13	0.269

Sexual activity was dichotomized into sexual activity (at least one sexual intercourse per week on average) versus no sexual activity (zero sexual intercourse per week on average). Values are percentages unless otherwise stated. ^a^ Effect size was calculated using phi coefficient and Cramer’s V for categorical variables and Cohen’s d for continuous variables. ^b^ *p*-values were based on chi-squared tests for categorical variables and on *t*-tests for continuous variables.

**Table 2:** Adjusted odds ratios (95% confidence interval) for prevalence of weekly sexual activity in relation to depression levels (reference subgroup: minimal).

	Model 1 ^a^ OR (95% CI)	Model 2 ^b^ OR (95% CI)	Model 3 ^c^ OR (95% CI)
Overall			
Minimal	1	1	1
Mild	0.41 (0.20–0.82)	0.41 (0.20–0.84)	0.34 (0.14–0.82)
Moderate	0.36 (0.16–0.78)	0.41 (0.18–0.94)	0.22 (0.08–0.58)
Severe	0.18 (0.04–0.83)	0.23 (0.05–1.12)	0.09 (0.01–0.61)

Depression estimated through Beck Depression Inventory II (BD-II): minimal (0–13 points), mild (14–19 points), moderate (20–28 points), and severe (29–63 points). OR Odds Ratio.; CI Confidence Interval. ^a^ Model 1 Adjusted for sex and age. ^b^ Model 2 Adjusted for Model 1 and socioeconomic variables (marital status, employment, annual household income, and place of living). ^c^ Model 3 Adjusted for Model 2, days of confinement, daily screen exposure, pre-COVID-19 confinement sexual activity, and health variables (smoking habit, usual alcohol consumption, physical conditions, physical symptoms).

**Table 3:** Adjusted odds ratios (95% confidence interval) for prevalence of weekly sexual activity in relation to anxiety levels (reference subgroup: minimal).

	Model 1 ^a^ OR (95% CI)	Model 2 ^b^ OR (95% CI)	Model 3 ^c^ OR (95% CI)
Overall			
Minimal	1	1	1
Mild	0.39 (0.22–0.71)	0.44 (0.24–0.81)	0.40 (0.19–0.84)
Moderate	0.54 (0.22–1.32)	0.64 (0.25–1.61)	0.75 (0.24–2.30)

Anxiety estimated through Beck Anxiety Inventory (BAI): minimal anxiety (0–7 points), mild anxiety (8–15 points), and moderate anxiety (16–25 points). OR Odds Ratio; CI Confidence Interval. ^a^ Model 1 Adjusted for sex and age. ^b^ Model 2 Adjusted for Model 1 and socioeconomic variables (marital status, employment, annual household income, and place of living). ^c^ Model 3 Adjusted for Model 2, days of confinement, daily screen exposure, pre-COVID-19 confinement sexual activity, and health variables (smoking habit, usual alcohol consumption, physical conditions, physical symptoms).

## Data Availability

The data that support the findings of this study are available from the corresponding authors upon reasonable request.

## References

[1] Gobierno de España (2020). Real Decreto 463/2020, de 14 de marzo, por el que se declara el estado de alarma para la gestión de la situación de crisis sanitaria ocasionada por el COVID-19. https://www.boe.es/eli/es/rd/2020/03/14/463.

[2] World Health Organization Mental Health and Psychosocial Considerations during the COVID-19 Outbreak, 18 March 2020. https://www.who.int/docs/default-source/coronaviruse/mental-health-considerations.pdf.

[3] Camuñas M.J.G. (2021). Qualitative analysis of the Spanish Health System in the context of COVID-19 pandemic. Atena J. Public Health.

[4] Jacob L., Smith L., Butler L., Barnett Y., Grabovac I., McDermott D., Armstrong N., Yakkundi A., Tully M.A. (2020). Challenges in the practice of sexual medicine in the time of COVID-19 in the United Kingdom. J. Sex. Med..

[5] Yuksel B., Ozgor F. (2020). Effect of the COVID-19 pandemic on female sexual behavior. Int. J. Gynecol. Obstet..

[6] Tan R.K.J., O’Hara C.A., Kumar N., Chow E. (2021). Partnership status, living arrangements, and changes in sexual behaviour and satisfaction during the COVID-19 lockdown: Insights from an observational, cross-sectional online survey in Singapore. Sexual Health.

[7] López-Bueno R., López-Sánchez G.F., Gil-Salmerón A., Grabovac I., Tully M.A., Casaña J., Smith L. (2021). COVID-19 Confinement and Sexual Activity in Spain: A Cross-Sectional Study. Int. J. Environ. Res. Public Health.

[8] Bach L.E., Mortimer J.A., VandeWeerd C., Corvin J. (2013). The association of physical and mental health with sexual activity in older adults in a retirement community. J. Sex. Med..

[9] Greenstein A., Abramov L., Matzkin H., Chen J. (2006). Sexual dysfunction in women partners of men with erectile dysfunction. Int. J. Impot. Res..

[10] Ganong K., Larson E. (2011). Intimacy and belonging: The association between sexual activity and depression among older adults. Soc. Ment. Health.

[11] Smith J.F., Breyer B.N., Eisenberg M.L., Sharlip I.D., Shindel A.W. (2010). Sexual function and depressive symptoms among male North American medical students. J. Sex. Med..

[12] Ministerio de Sanidad Orden SND/399/2020, de 9 de mayo, para la flexibilización de determinadas restricciones de ámbito nacional, establecidas tras la declaración del estado de alarma en aplicación de la fase 1 del Plan para la transición hacia una nueva normalidad. https://www.boe.es/eli/es/o/2020/05/09/snd399/con.

[13] Vandenbroucke J.P., von Elm E., Altman D.G., Gøtzsche P.C., Mulrow C.D., Pocock S.J., Poole C., Schlesselman J.J., Egger M. (2014). STROBE Initiative Strengthening the Reporting of Observational Studies in Epidemiology (STROBE): Explanation and elaboration. Int. J. Surg..

[14] Sanz J. (2014). Manual BAI. Inventario de Ansiedad de Beck (adaptación española de J. Sanz).

[15] Magán I., Sanz J., García-Vera M.P. (2008). Psychometric properties of a Spanish version of the Beck Anxiety Inventory (BAI) in general population. Span. J. Psychol..

[16] Sanz J., Perdigón A.L., Vázquez C. (2003). Adaptación española del Inventario para la Depresión de Beck-II (BDI-II): 2. Propiedades psicométricas en población general. Clínica Salud.

[17] Vázquez C., Sanz J. (1997). Fiabilidad y valores normativos de la versión española del inventario para la depresión de Beck de 1978. Clínica Salud.

[18] Beck A.T., Ward C.H., Mendelson M., Mock J., Erbaugh J. (1961). An inventory for measuring depression. Arch. Gen. Psychiatry.

[19] Ministry of Health of the Kingdom of Spain (2017). National Survey of Health 2017. https://www.mscbs.gob.es/estadEstudios/estadisticas/encuestaNacional/encuesta2017.htm.

[20] Ibarra F.P., Mehrad M., Mauro M.D., Godoy M.F.P., Cruz E.G., Nilforoushzadeh M.A., Russo G.I. (2020). Impact of the COVID-19 pandemic on the sexual behavior of the population. The vision of the east and the west. Int. Braz J. Urol..

[21] Lehmiller J.J., Garcia J.R., Gesselman A.N., Mark K.P. (2021). Less sex, but more sexual diversity: Changes in sexual behavior during the COVID-19 coronavirus pandemic. Leis. Sci..

[22] Arafat S.Y., Alradie-Mohamed A., Kar S.K., Sharma P., Kabir R. (2020). Does COVID-19 pandemic affect sexual behaviour? A cross-sectional, cross-national online survey. Psychiatry Res..

[23] Li W., Li G., Xin C., Wang Y., Yang S. (2020). Challenges in the practice of sexual medicine in the time of COVID-19 in China. J. Sex. Med..

[24] Enríquez-Reyna M.C., Gurrola O.C., Rodríguez R.E.M., Cocca A., Muciño O.M. (2021). Características de la práctica de actividad física durante pandemia por COVID-19 en profesionistas y sus familias. SPORT TK-Rev. EuroAmericana De Cienc. Del Deporte.

[25] López-Bueno R., Calatayud J., Ezzatvar Y., Casajús J.A., Smith L., Andersen L.L., Lopez-Sanchez G.F. (2020). Association between current physical activity and current perceived anxiety and mood in the initial phase of COVID-19 confinement. Front. Psychiatry.

[26] Jacob L., Tully M.A., Barnett Y., Lopez-Sanchez G.F., Butler L., Schuch F., López-Bueno R., McDermott D., Firth J., Grabovac I. (2020). The relationship between physical activity and mental health in a sample of the UK public: A cross-sectional study during the implementation of COVID-19 social distancing measures. Ment. Health Phys. Act..

[27] Rodríguez-Rey R., Garrido-Hernansaiz H., Collado S. (2020). Psychological impact and associated factors during the initial stage of the coronavirus (COVID-19) pandemic among the general population in Spain. Front. Psychol..

[28] Brooks S.K., Webster R.K., Smith L.E., Woodland L., Wessely S., Greenberg N., Rubin G.J. (2020). The psychological impact of quarantine and how to reduce it: Rapid review of the evidence. Lancet.

[29] Mahanty C., Kumar R., Mishra B.K. (2021). Analyses the effects of COVID-19 outbreak on human sexual behaviour using ordinary least-squares based multivariate logistic regression. Qual. Quant..

